# The Value of Dynamic MRI in Cervical Radiculopathy: A Report of Two Cases

**DOI:** 10.7759/cureus.83321

**Published:** 2025-05-01

**Authors:** Bhavika Gupta, Dallas Kramer, Caitlin C Barrett, Chen Xu, Alexander Yu

**Affiliations:** 1 Neurological Surgery, Allegheny Health Network, Pittsburgh, USA; 2 Neurosurgery, Drexel College of Medicine, Philadelphia, USA; 3 Neurosurgery, Allegheny General Hospital, Pittsburgh, USA

**Keywords:** dynamic imaging, extension, flexion, myelopathy, radiculopathy

## Abstract

Dynamic imaging is well established for evaluating cervical myelopathy but is less commonly utilized for cervical radiculopathy. Typically, cervical radiculopathy is diagnosed using static imaging modalities such as CT or MRI. However, recent studies have demonstrated that spinal disorders can be dynamic in nature and may not be fully captured with static imaging alone. Dynamic MRI (dMRI), performed with the neck in flexion and extension, can assist both in diagnosis and surgical planning. We report two cases of cervical radiculopathy with persistent symptoms despite normal findings on standard static imaging. A dMRI sequence was performed to better visualize the underlying pathology. Notably, movement-dependent changes in the width of the spinal canal were observed. Both patients subsequently underwent artificial disc arthroplasty and experienced complete resolution of their symptoms. While dMRI is not necessary for all patients with spinal conditions, it may offer significant diagnostic value in those with persistent symptoms and inconclusive static imaging. Additionally, it can help clarify spinal anatomy and enhance the precision of surgical planning.

## Introduction

Cervical radiculopathy is among the most common presenting complaints in spine clinics, with an annual incidence of 0.8 per 1,000 individuals [1]. This condition results in significant cumulative healthcare costs even before surgical intervention, which is typically reserved for patients who do not respond to conservative treatment [2]. While physical examination maneuvers such as Spurling’s test rely on dynamic nerve root compression at the neuroforamina to aid diagnosis, radiographic evaluation remains largely static.

Static or neutral imaging may be insufficient to identify complex biomechanical abnormalities of the spine. Several studies have shown that spinal cord disorders are often dynamic rather than static in nature [3,4]. Dynamic MRI (dMRI), which captures images during flexion and extension of the cervical spine, allows for the assessment of mechanical changes and reveals the impact of intervertebral discs and ligaments on neural structures [5].

Although dMRI has been explored in the context of degenerative cervical myelopathy, its application in patients with foraminal stenosis or cervical radiculopathy remains unreported in the literature [5]. The diagnostic value of dMRI in identifying cervical radiculopathy, particularly when caused by foraminal stenosis that is not clearly visible on static imaging, has yet to be established. Given the significant impact of neck pain and radicular symptoms on quality of life, it is essential to develop a diagnostic approach for patients who exhibit persistent symptoms despite normal or unremarkable static imaging and who have not improved with conservative measures. For such patients, well-planned cervical spine surgery based on dynamic imaging findings could lead to substantial improvements in pain and quality of life.

In this report, we present two cases of cervical radiculopathy in which dynamic cervical MRI revealed disc herniations that were not visible on neutral static imaging. At our institution, dMRI was performed by positioning patients in maximum neck flexion and extension without discomfort. During maximum extension, a prop was placed between the shoulder blades for support; during maximum flexion, a prop was positioned behind the neck. Based on the dMRI findings, both patients underwent cervical disc arthroplasty and achieved complete symptom resolution. These cases demonstrate the potential benefit of dMRI for patients with persistent symptoms but unremarkable static imaging, allowing for more precise surgical planning based on the patient’s anatomy in motion.

## Case presentation

Case 1

**Figure 1:**
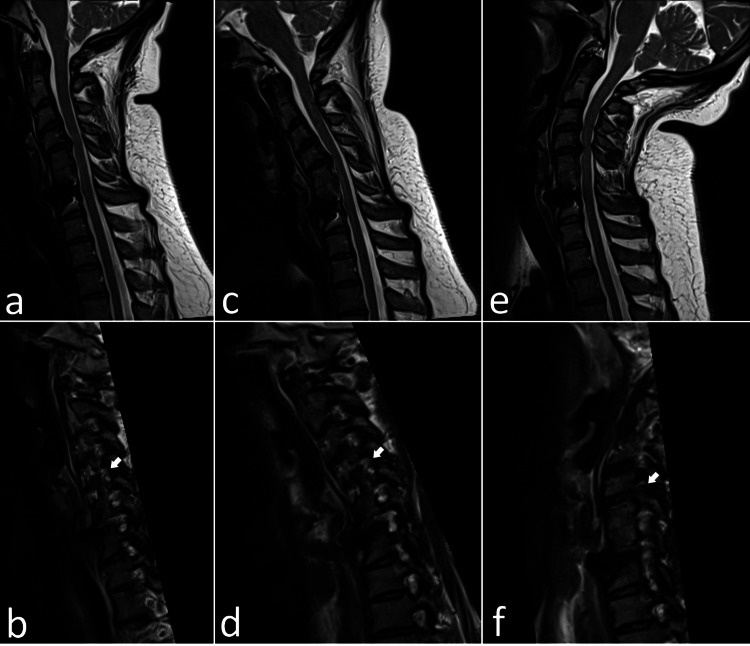
T2-weighted MRI images of the cervical spine in sagittal and right parasagittal views during (a, b) neutral position, (c, d) flexion, and (e, f) extension. In the extended position, there is increased disc protrusion along with posterior ligamentous buckling, resulting in severe spinal canal stenosis at the C4-C5 level. Neck extension also leads to marked stenosis of the right C4-C5 neuroforamen (arrows).

A 41-year-old female with a history of C5-C6 anterior cervical discectomy and fusion (ACDF) and C6-C7 disc arthroplasty presented with two years of right-sided neck, shoulder blade, and proximal arm pain, along with paresthesias following a motor vehicle collision. On physical examination, she exhibited isolated right deltoid weakness graded 4+/5 and numbness across the right shoulder blade. An MRI of the cervical spine revealed a broad-based C4-C5 disc osteophyte complex with moderate central canal stenosis and no significant foraminal stenosis (Figure 1a). Cervical flexion-extension radiographs showed no evidence of instability. Her symptoms were refractory to conservative management, including right C5 and C6 selective nerve root blocks.

A repeat MRI of the cervical spine with flexion and extension was obtained. The anterior-posterior diameter of the spinal canal was relatively preserved between the neutral position (Figure 1a) and flexion (Figure 1c), measuring 8.7 mm and 8.3 mm, respectively. However, with extension (Figure 1e), the spinal canal diameter decreased to 5.3 mm due to ligamentum flavum buckling and an increased broad-based central disc protrusion. Additionally, with neck extension, significant stenosis of the right C4-C5 neuroforamina (Figure 1f) was observed, which was not present in the neutral (Figure 1b) or flexed (Figure 1d) positions.

She underwent a C4-C5 disc arthroplasty, resulting in immediate pain resolution and improvement in right deltoid weakness to 5/5 strength, which was maintained at her most recent three-month follow-up.

Case 2

A 51-year-old female presented with nine months of right-sided neck pain accompanied by paresthesias radiating into the lateral arm, forearm, and first three digits. Her symptoms were transient and provoked by neck extension. Notably, she had a remote history of thoracic outlet syndrome and had undergone a right first rib resection 12 years prior. On physical examination, she had 5/5 strength in all muscle groups, normal sensation without hyperesthesia, 2+ biceps and brachioradialis reflexes, and a positive right Spurling’s test. Her symptoms were refractory to conservative management, which included non-steroidal anti-inflammatory drugs, oral steroids, physical therapy, and chiropractic manipulation.

**Figure 2:**
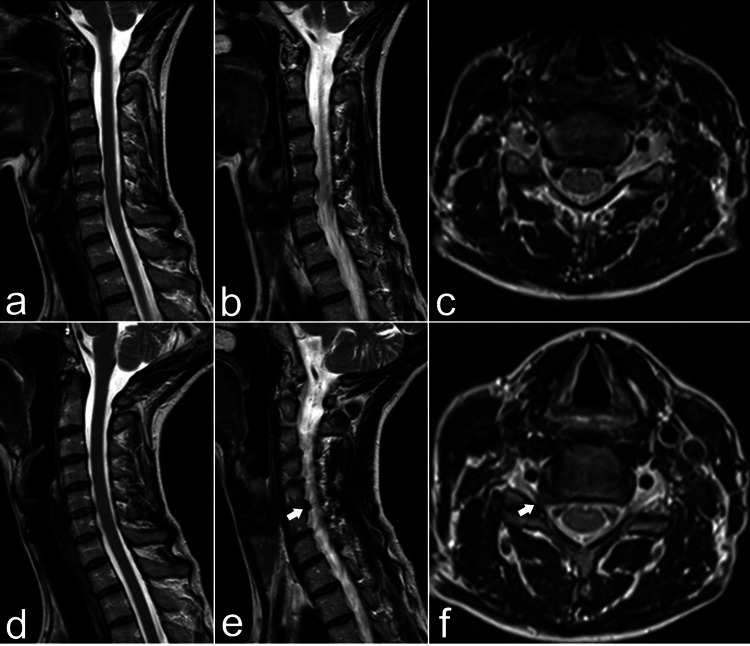
T2-weighted MRI in the neutral position: (a) sagittal, (b) right parasagittal, and (c) T1.5-weighted axial images, with corresponding extension images shown in (d-f). In the neutral position, there is no significant spinal canal or foraminal stenosis. However, with neck extension, a prominent disc protrusion is observed, resulting in severe right 6 foraminal stenosis (arrow).

MRI of the cervical spine showed mild degenerative disc changes at C5-C6 and C6-C7, with no significant central canal or foraminal stenosis (Figure 2a-2c). Cervical flexion-extension radiographs showed no instability, and CT myelogram demonstrated mild C5-C6 and C6-C7 central canal stenosis, along with mild left C5-C6 foraminal stenosis. Given her history, an MRI of the brachial plexus was obtained, which only revealed postoperative changes from the right first rib resection. EMG/NCV studies showed no evidence of radiculopathy, myopathy, peripheral neuropathy, or compressive neuropathy.

A repeat MRI of the cervical spine with flexion and extension sequences was performed. Sagittal extension T2 MRI showed an increased prominence of the disc osteophyte complex at the right C5-C6 uncovertebral joint, resulting in severe right C5-C6 foraminal stenosis (Figure 2d-2f). The patient underwent C5-C6 disc arthroplasty, with immediate resolution of her preoperative symptoms, which were sustained at the four-month follow-up.

## Discussion

Cervical radiculopathy results from nerve root compression as it exits the foramina. This impingement can arise from various causes, including degenerative joint disease, trauma, or spinal lesions. Age-related disc and bone degeneration can alter the bone-nerve interface and contribute to foraminal narrowing, ultimately leading to radicular symptoms. Understanding changes in the neural foramen dimensions is essential for grasping the pathogenesis of cervical radiculopathy and for effectively planning decompressive surgery.

This study highlights the utility of dMRI in detecting dynamic disc herniation in patients who show no significant degenerative changes on CT, instability on dynamic radiographs, or foraminal stenosis on static MRI. In these cases, disc herniation correlated with the patients’ radicular symptoms was only evident in dMRI extension sequences. Neither patient had sufficient foraminal stenosis on static imaging to warrant ACDF or posterior cervical foraminotomy.

Kitagawa et al. demonstrated that spinal dimensions change significantly with movement in healthy individuals: flexion increased the foraminal area by 28%, while extension decreased it by 17% compared with the neutral position [6]. They also noted that although dynamic CT effectively captures bony changes, it may fall short in detecting soft tissue alterations surrounding the foramen. Farmer and Wisneski further concluded that neck extension increases pressure on foraminal nerves, potentially worsening radicular pain [7]. Soft tissue compression and ischemic processes around the foramen are also known contributors to cervical radiculopathy. While CT is valuable for evaluating bony structures, it lacks sensitivity for surrounding soft tissues, possibly leading to missed diagnoses [7].

The value of dynamic imaging for spinal pathologies was first noted by Epstein in 1998 [8]. Since then, numerous cadaveric and in vivo studies have shown significant changes in spinal canal and foraminal dimensions with flexion and extension. Matsunaga et al. suggested that neck extension may cause the spinal cord to drape over osteophytes, worsening cervical myelopathy [9], while Endo et al. postulated that extension shortens the spinal cord and increases pressure on exiting nerves, exacerbating radiculopathy [10]. A recent review involving 2,662 patients found that spinal canal diameter decreased during extension [5].

Several studies have questioned the diagnostic accuracy of static imaging for spinal disorders. Static MRI often has low specificity for diagnosing cervical radiculopathy [11]. While severe stenosis may be captured on static MRI, mild to moderate cases can go undetected. Muhle et al. studied 81 patients with dMRI and found that neck extension in those with degenerative disc disease and ligamentum flavum hypertrophy reduced canal diameter and worsened symptoms [12]. Dalbayrak et al. similarly observed a 13.4% decrease in spinal canal diameter during extension [3].

For patients whose symptoms are not explained by initial imaging, myelography has traditionally been the next diagnostic step. While CT myelography mimics neck extension to some degree, it is invasive, time-consuming, exposes patients to radiation, and provides limited soft tissue visualization. In contrast, extension dMRI in a supine 1.5T scanner can achieve similar positional extension while maintaining superior visualization of soft tissues and bones [13]. dMRI has also been useful in surgical planning for patients with cervical instability caused by conditions such as rheumatoid arthritis, os odontoideum, and achondroplasia [5]. Bartlett et al. compared dMRI to spinal myelography and found that dynamic imaging, whether MRI or myelography, was more helpful than static MRI in diagnosing foraminal stenosis and guiding surgical decisions in patients with radiculopathy [14].

Patients often report that certain positions or movements worsen their symptoms. Since maximum stress on the cervical spine occurs in the upright position, imaging under these conditions can reveal a more accurate picture of pathology [5]. Modern MRI systems allow for neck flexion and extension, making dynamic imaging more accessible. Jinkins et al. used a modified MRI system with a tilt table to study spinal changes dynamically. Their study found that static MRI underestimated disc herniation and degenerative changes and that kinematic MRI offered a more comprehensive view of dynamic spinal pathologies [15].

Overall, the enhanced visualization of cervical spine pathology offered by dynamic imaging significantly improves surgical planning without adding cost or inconvenience to the patient. Surgical care should be tailored to each patient’s specific anatomy, and dynamic imaging can be an essential step in that direction. While larger, multicenter studies are needed to support routine dMRI use in cervical radiculopathy, it is a valuable tool in patients with persistent symptoms and inconclusive static imaging.

Both patients in our study had ongoing symptoms of cervical radiculopathy despite unremarkable static imaging. Their underlying pathology was identified only on dMRI, and both experienced complete symptom resolution following cervical disc arthroplasty. As a biomechanical condition, cervical radiculopathy is often inadequately assessed using static CT or MRI, which have been shown to lack sensitivity. dMRI using extension and flexion sequences can help diagnose cases missed by conventional imaging and assist in planning more precise surgeries. Future multicenter studies with larger cohorts of symptomatic patients could help standardize the use of dMRI in the diagnostic workup of cervical radiculopathy.

## Conclusions

dMRI serves as a valuable diagnostic tool for evaluating cervical radiculopathy in patients with persistent symptoms despite normal findings on static imaging. The cases presented here illustrate how dMRI can reveal dynamic disc herniations and foraminal stenosis that might otherwise go undetected. In Case 1, although standard cervical flexion-extension radiographs showed no evidence of instability, dMRI revealed significant stenosis of the right C4-C5 neuroforamina, correlating precisely with the patient’s symptoms. Similarly, in Case 2, dMRI identified a compressive disc osteophyte complex that was not clearly visible on prior standard radiographs or static MRI. Based on these dMRI findings, both patients underwent cervical disc arthroplasty and experienced significant symptom relief. By capturing real-time biomechanical changes, dMRI deepens our understanding of cervical spine pathology and offers a promising avenue to refine current diagnostic and therapeutic strategies. Further research with larger patient cohorts is needed to establish standardized protocols and explore the broader clinical utility of dMRI in conditions like cervical myelopathy.
